# 

**Published:** 1875-09

**Authors:** 


### Deaths.

MAYNARD. August 20, at her residence, 130 Loomis street, after a protracted and painful illness, Maria M., wife of Dr. Wm. J. Maynard.

---

### ANNOUNCEMENTS FOR THE MONTH.

### MONDAYS.

SOCIETIES.

*Mondays, Sept.* 6th and 20th—Chicago Medical Society, regular meetings at Gault House, 8 P. M.

*Mondays, Sept.* 13th and 27th—Chicago Association of Physicians and Surgeons, regular meetings at Pacific Hotel, 8 P. M.

CLINICS.

*Every Monday.* At Illinois Charitable Eye and Ear Infirmary (Peoria and Adams Sts.) 2 P. M. Prof. Holmes.

At Chicago Medical College, 2 P. M. *Gynecological*—Dr. Merriman.

At Central Dispensary (239 W. Van Buren St.) 2 P. M., *Gynecological*—Dr. Adolphus; 3 P. M., *Medicine*—Dr. Bridge.

### TUESDAYS.

CLINICS. *Every Tuesday.*

At Cook County Hospital, 3 P. M., *Surgical*—Prof. Freer.

At Chicago Medical College, 2 P. M., *Gynecological*—Dr. Roler.

### WEDNESDAYS.

CLINICS. *Every Wednesday.*

At Chicago Medical College, 2 P. M., *Gynecological*—Dr. Nelson.

At "Woman's Dispensary," (229 30th St.,) 11 A. M., *Gynecological*—Drs. Hurlbut and Jackson; 1 P. M., *Electrical*—Dr. P. S. Hayes.

At Central Dispensary, 3 P. M. *Medical*—Dr. Bridge.

### THURSDAYS.

CLINICS. *Every Thursday.*

At the Illinois Charitable Eye and Ear Infirmary, 2 P. M., Prof. Holmes.

At Chicago Medical College, 2 P. M., *Gynecological*—Dr. Merriman.

At Central Dispensary, 2 P. M., *Gynecological*—Dr. Adolphus.

### FRIDAYS.

CLINICS. *Every Friday.*

At Cook County Hospital, 3 P. M., *Surgical*—Prof. Freer.

At Chicago Medical College, 2 P. M., *Gynecological*—Dr. Roler.

### SATURDAYS.

CLINICS. *Every Saturday.*

At "Woman's Dispensary," 11 A. M., Drs. Hurlbut and Jackson; at 1 P. M., Dr. P. S. Hayes.

At Chicago Medical College, 2 P. M., *Gynecological*—Dr. Nelson.

